# Sonde de Dormia enclavée dans le cholédoque: une complication peu connue

**DOI:** 10.11604/pamj.2021.38.82.27576

**Published:** 2021-01-25

**Authors:** Khalil Maamar, Mohammed Bouziane

**Affiliations:** 1 Université Mohammed VI, Chirurgie Générale A, Oujda, Maroc

**Keywords:** Lithiase, Dormia, angiocholite, choledocotomie, Lithiasis, Dormia, angiocholitis, choledocotomy

## Abstract

Conventional treatment of lithiasis of the main biliary tract (MBT) is based on endoscopic sphincterotomy followed by extraction of the stone(s) using a balloon catheter and/or Dormia catheter. Several risk factors associated with failure to remove a stone in the main biliary tract have been reported. The most important factor is probably a stone volume greater than 15 mm or an initial diameter of the MBT smaller than that of the stone. Care must be taken not to impact the stone in the Oddi sphincter or in the Dormia in order not to compromise stone removal. Complications associated with the use of the Dormia catheter include bile duct injuries, which are a well-known surgical problem. Injuries specifically related to the use of catheter have been less reported and seem to be underestimated. We here display an image showing Dormia catheter trapped in the bile duct around a fixed stone which couldn´t be removed after endoscopic exploration in a 60-year-old patient with cholangitis caused by lithiasis. Choledochotomy for removal of stone was performed.

## Image en médecine

Le traitement conventionnel de la lithiase de la voie biliaire principale (VBP), consiste en une sphinctérotomie endoscopique suivie d´une extraction du ou des calculs au ballon et/ou à l´anse de Dormia. Plusieurs facteurs de risques d´échec d´extraction d´un calcul de la voie biliaire principale sont connus. Le facteur probablement le plus important est la taille du calcul supérieur à 15 mm ou lorsque le diamètre du bas de la VBP est inférieur à celui du calcul où il faut veiller à ne pas impacter le calcul dans le sphincter d´Oddi ou dans la Dormia afin de ne pas compromettre la suite de l´extraction. Parmi les complications liées à l'utilisation de la sonde de Dormia, les plaies biliaires, constituent un problème bien connu des chirurgiens, mais les lésions spécifiquement liées à l'utilisation de la sonde le sont moins et paraissent sous-estimées. Nous rapportons une image où la sonde de Dormia est restée enclavée dans le choledoque autour d´un calcul qui est resté lui-même fixé sans possibilité de la retirer après exploration endoscopique chez un patient de 60 ans en angiocholite lithiasique nous obligeant à faire une extraction chirurgicale par choledocotomie.

**Figure 1:**
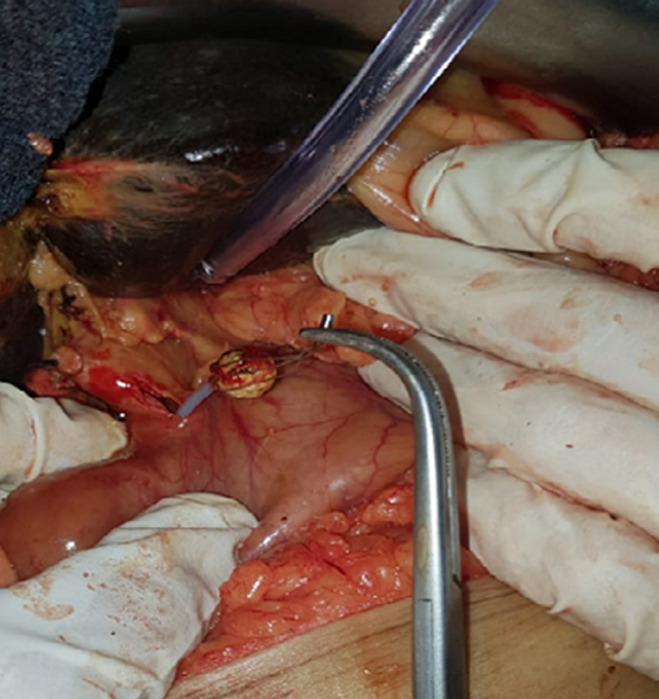
la sonde de Dormia, restée enclavée dans le choledoque

